# Lenalidomide-Associated Immune Thrombocytopenia: A Case Report and Review of the Literature

**DOI:** 10.1155/2020/8825618

**Published:** 2020-11-12

**Authors:** William Forehand III, Germame Ajebo, Michael Toscano, Anand Jillella, Paul Dainer

**Affiliations:** ^1^Division of Hematology-Oncology, Medical College of Georgia, Augusta University, Georgia Cancer Center, Augusta, Georgia; ^2^Department of Pathology and Laboratory Medicine, Augusta University, Augusta, Georgia

## Abstract

Lenalidomide is indicated in the front-line management of multiple myeloma. More recently, it has been introduced for use in treating other hematologic malignancies. Although the drug is known to cause myelosuppression, there have been rare reports of lenalidomide-associated immune thrombocytopenia (ITP). Here, we review the literature on lenalidomide-associated ITP and report upon a 59-year-old man who was administered lenalidomide due to concern of progressive multiple myeloma more than a year following his having undergone an autologous hematopoietic stem cell transplant. His platelet count precipitously declined and lead to his hospitalization. Despite our withholding of the drug, he did not respond to platelet transfusions or administration of corticosteroids. He was successfully managed with intermittent immune globulin for several months before definitive treatment with splenectomy, which resulted in the complete resolution of his thrombocytopenia. A literature search identified a total of six additional cases of lenalidomide-associated ITP. Similarly, many of the reported cases were associated with persistent thrombocytopenia after discontinuation of the drug. Furthermore, these patients were generally managed successfully with standard ITP therapies, such as corticosteroids or intravenous immune globulin.

## 1. Introduction

Immune thrombocytopenia (ITP) is a disorder characterized by immune-mediated thrombocytopenia [1]. Idiopathic ITP is reportedly more common; however, ITP secondary to underlying conditions or drugs has been identified not infrequently and may be underdiagnosed [2–4]. Several subtypes of drug-induced ITP (DITP) have been described based on their proposed mechanisms: hapten-dependent, drug-dependent, fiban-induced, drug-specific, autoantibody, and immune complex.

In most circumstances, there must be ongoing exposure to the offending drug for the thrombocytopenia to persist [5]. Hence, the mainstay of managing DITP has been to discontinue the drug whenever possible. Furthermore, criteria have been proposed by George and others for assessing possible DITP cases [6]. Based on such criteria, a case of definitive DITP would require that the patient's thrombocytopenia recovers after the drug is discontinued; however, this definition would exclude cases of DITP of the autoantibody subtype from being considered definite DITP. In the autoantibody subtype of DITP, thrombocytopenia can persist following the removal of the offending drug. The mechanism of this subtype of DITP is thought to involve the production of autoantibodies to platelets that do not depend upon the presence of the drug for binding, such as in primary ITP. Autoantibody-type DITP has been reported with gold and sulfamethoxazole [5, 7].

There have been very few cases of lenalidomide-associated ITP reported in the literature [8–10]. Of these cases, several have been reported in which the thrombocytopenia persisted after stopping the lenalidomide [10]. Here, we report another distinct case of lenalidomide-associated ITP that persisted for months after stopping this agent, possibly placing it among the drugs which can cause the autoantibody subtype of DITP.

## 2. Case Presentation

The patient was a 59-year-old male who had been diagnosed with plasma cell myeloma in 2004 when he presented with pain and lytic lesions on a skeletal survey and an associated monoclonal IgG on a serum protein electrophoresis (SPEP) and immunofixation. A bone marrow biopsy demonstrated 30% lambda-restricted plasma cells at that time. He was treated with dexamethasone and thalidomide and achieved a very good partial response prior to receiving high-dose melphalan and underwent an autologous peripheral hematopoietic stem cell transplant (auto-HSCT) in July 2005. He achieved a complete response as a bone marrow aspirate, and biopsy in January 2006 revealed less than five percent polyclonal plasma cells. By the following month, his platelet count had recovered to 208,000/mm^3^.

Following his recovery, the patient's care was transitioned back to his local hospital. In September 2006, he experienced increasing hip pain. Radiographic imaging revealed bilateral lesions in the femoral necks and pelvis. A bone marrow aspirate and biopsy at that time demonstrated maturing trilineage hematopoiesis and no monoclonal plasma cell population. An SPEP on September 25, 2006, demonstrated a rising M-spike of 0.65 g/dL. In October 2006, he underwent a right hip nail procedure to avoid an impending fracture. The pathologic specimen retrieved from that surgery showed fragments of bone with necrosis and interspersed poorly preserved crushed cells, which could not be definitively characterized.

By November 2006, his M-spike further increased to 0.77 g/dL. Based on the bone lesions and an increasing M-spike, a decision was made to initiate an attenuated course of lenalidomide and dexamethasone, along with a low dose of warfarin. The patient's transplant physician had recommended a 21-day 10 mg daily dose, which was initiated on December 30, 2006. He also received 40 mg dexamethasone every two weeks. No other new medications were initiated at that time. His chronic medications included metformin, glipizide, atenolol, pamidronate aspirin, fosinopril, gabapentin, lansoprazole, simvastatin, and ezetimibe. On January 26, 2007, the patient was admitted to the hospital for severe thrombocytopenia with a platelet count of 9,000/mm^3^; his other blood counts at the time of admission included Hgb, 10.7 g/dL, which was similar to his Hgb level before initiating lenalidomide. His WBC count was normal at 5.8 k/mm^3^. Further lenalidomide was withheld, and he was administered a 3-day course of 1 mg/kg prednisone along with a platelet transfusion; however, there was no increment in his platelet count. His HIV, hepatitis B, hepatitis C, and ANA screens were negative. On February 5, 2007, he underwent an abdominal ultrasound which demonstrated no evidence of portal hypertension or splenomegaly. Subsequently, he was transferred to the Augusta University Medical Center. At the time of transfer on February 6, 2007, his platelet count was 17,000/mm^3^.

**Figure 1:**
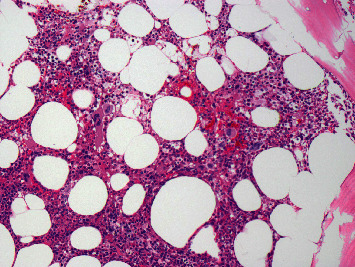
Bone marrow biopsy (200x). The bone marrow is normocellular with adequate trilineage hematopoiesis and normal maturation. Several megakaryocytes are present.

Following his transfer, he received transfusions of several more units of platelets on February 8^th^ and 9^th^, but his platelets continued to decline and fell to a nadir of 5,000/mm^3^ on February 9th. A bone marrow aspirate and biopsy were performed. The specimens obtained demonstrated maturing hematopoiesis with adequate megakaryopoiesis and no morphologic evidence of recurrent myeloma (Figure 1). His hepatic function tests and renal function remained normal throughout his clinical course. In the absence of abnormal bleeding, he was discharged and maintained under close observation as an outpatient.

**Figure 2:**
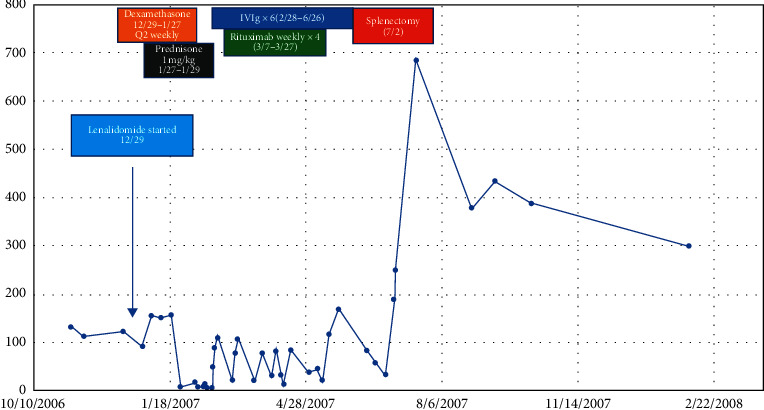
The platelet count and treatment history of the case.

**Figure 3:**
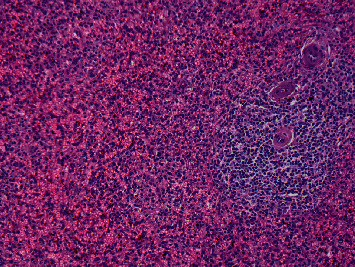
The spleen was 244.5 gm showing red pulp congestion, but no abnormal cell population. The perivascular white pulp is intact but attenuated.

Despite having had the lenalidomide withheld, his thrombocytopenia persisted, and he was readmitted to the hospital on February 18, 2007, after the onset of epistaxis, petechiae over his ankles, and melenic stools. He received intravenous gamma globulin (IVIG) on February 18^th^ and achieved a rapid and robust platelet response. His platelets increased to 48,000/mm^3^ on the following day and peaked at 110,000/mm^3^ on February 22^nd^. The response was not sustained, and his platelets again decreased to 19,000/mm^3^. He received an additional dose of IVIG, which again resulted in a transient response. A course of rituximab was added to the treatment regimen. He remained dependent on intermittent IVIG infusions until July 2, 2007, when he underwent a splenectomy, resulting in a sustained rise in his platelet count (Figure 2). His spleen had a normal weight of 244.5 gm, and microscopic examination demonstrated red pulp congestion and no abnormal cell populations (Figure 3). Within a few months, he developed new bone lesions with an associated hip fracture, and his serum paraprotein increased further. He was treated sequentially with thalidomide and prednisone and, later, bortezomib, liposomal doxorubicin, and dexamethasone with symptomatic improvement and no recurrence of significant thrombocytopenia. He was lost to follow-up after a year of treatment and reportedly expired of uncertain causes.

## 3. Methods

**Figure 4:**
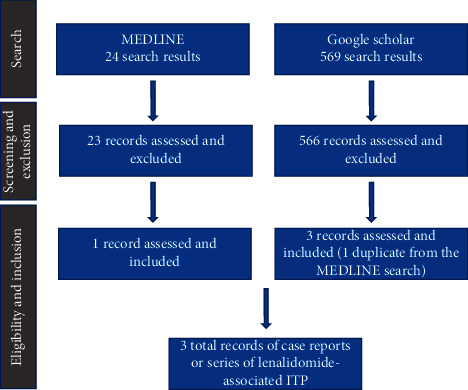
Literature search results, exclusion, and inclusion. A MEDLINE search for the keywords (lenalidomide) and (ITP) or (lenalidomide) and (immune thrombocytopenia) resulted in 24 results. 23 of these results were excluded, and 1 was assessed and included. Google Scholar was also searched using the keywords (lenalidomide) and (ITP). There were 569 results. 566 of these results were assessed and excluded as not related to lenalidomide and ITP. 3 of the 569 results were included in our report. Because one of the included Google Scholar results was a duplicate with the included MEDLINE, there were a total of 3 reports that were identified that those were case reports or case series of lenalidomide-associated ITP.

Using PubMed and Ovid, we searched the MEDLINE database using a date range from 1980–2020 for the keywords (lenalidomide) and (ITP) or (lenalidomide) and (immune thrombocytopenia). There were 24 search results. Only one result reported on a case of lenalidomide-associated ITP. A separate search using Google Scholar was conducted, using the keywords (lenalidomide) and (ITP). There were 569 search results. There were two additional articles or abstracts that reported on cases of lenalidomide-associated ITP. A summary of the search results is shown in Figure 4.

## 4. Discussion

Lenalidomide is a drug supported by national guidelines for the front-line management of plasma cell myeloma [11]. More recently, the U.S. Food and Drug Administration (FDA) approved the use of lenalidomide as maintenance therapy after auto-HSCT for patients with myeloma. The approval was based on evidence from two randomized, blinded trials of maintenance lenalidomide versus placebo [12]. One of its well-known toxicities has been reversible myelosuppression; however, there have only been rare reports of DITP from lenalidomide [8–10]. Our MEDLINE and Google Scholar search only identified three reports with a total of six cases of lenalidomide-associated ITP (summarized in Table 1).

Most cases of DITP related to drugs other than lenalidomide have been associated with platelet recovery after stopping the drug. Improvement in platelet counts after removing exposure to the offending drug establishes drug dependence of the immune-mediated platelet destruction. Such cases, especially with recurrence of the thrombocytopenia upon re-exposure to the drug, can be considered definitive DITP by criteria proposed by George and others [6]; however, such criteria do not capture those few cases characterized by autoantibodies able to destroy platelets without depending upon the presence of the inciting drug. Such cases of DITP can be associated with ongoing thrombocytopenia that resembles primary ITP. Here, we reported a case of secondary ITP associated with lenalidomide exposure that persisted after stopping lenalidomide for several months, long after its biotransformation and rapid renal clearance. Our case suggests that lenalidomide may be able to induce persistent immune-mediated platelet destruction. While cases of thrombocytopenia resolution after discontinuation of lenalidomide have been reported, our case is consistent with the report of Pompa and colleagues [10]. They describe four cases of lenalidomide-associated ITP (Table 1). These cases were also associated with persistent thrombocytopenia or ITP-therapy dependence after discontinuation of lenalidomide. In one case, thrombocytopenia was successfully treated with steroids, but immune thrombocytopenia recurred after the patient resumed lenalidomide. In a case reported by Meguri and colleagues, a patient with lenalidomide-associated ITP also developed a rash and proteinuria (Table 1). The authors implicated a broader immunologic dysregulation as the underlying mechanism of lenalidomide-associated ITP [9].

In conclusion, lenalidomide may be an underrecognized cause of the autoantibody subtype of DITP resulting from the induction of nondrug-dependent autoantibody-mediated platelet destruction. As in primary ITP, our patient was successfully managed with IVIG and splenectomy. To our knowledge, this is the first case to report the successful treatment of lenalidomide-associated ITP with splenectomy. Our case, along with the other cases reported in the literature, suggests that the management of lenalidomide-associated ITP may require both drug discontinuation and therapies traditionally used in primary ITP. Splenectomy may be a successful approach to attain a durable response in patients who otherwise remain steroid-refractory or IVIG-dependent; however, it should be noted that thrombopoietin receptor agonists were not clinically available during that time and should be considered in recalcitrant cases. Our report and those of others should alert the clinician to this unusual mechanism of lenalidomide toxicity as this drug is currently being utilized in the treatment of an increasing number of malignant hematologic neoplasms.

## Figures and Tables

**Table 1:** A comparison of the cases of lenalidomide-associated ITP reported in the literature along with our case.

	Case 1[10]	Case 2 [10]	Case 3 [10]	Case 4 [10]	Case 5 [8]	Case 6 [9]	Our case
Age	66	76	78	66	27	74	59

Sex	F	F	F	F	M	F	M

Plasma cell dyscrasia subtype	IgGk MM	IgG*λ* MM	IgA*λ* amyloid	MM (subtype not reported)	MM (subtype not reported)	Light chain *λ* MM	IgG*λ* MM

Dose of len	15 mg	15 mg	15 mg	25 mg	25 mg	15 mg	10 mg

Previous autologous bone marrow transplant	No	No	No	No	Yes	No	Yes

Cycle of lenalidomide before developing thrombocytopenia	5	3	6	3	Received consolidation × 4 VRD and maintenance lenalidomide × 3 months	1	1

Treatment of ITP	Steroids, IVIg, rituximab	Steroids	Steroids	Steroids (prednisone)	Steroids and IVIG	Steroids	Steroids, IVIg, rituximab, splenectomy

Persistent thrombocytopenia after stopping lenalidomide	Yes	Yes, until steroids initiated. Responded to steroids at around 1 month after stopping lenalidomide	Yes, patient remained steroid-dependent	Yes	No, responded to steroid tapering	No, responded to steroid tapering	Yes, patient eventually obtained a long-term remission with splenectomy

Retreatment with lenalidomide was associated with recurrence of thrombocytopenia	Yes	Retreated, but no recurrent thrombocytopenia	Not retreated	Not retreated	Not retreated	Not retreated	Not retreated

Other manifestations	None reported	None reported	None reported	None reported	Alopecia, leukopenia	Rash, proteinuria	None

## Data Availability

The data used to support the findings of this study are included within the manuscript.
